# Swapping White for High-Fibre Bread Increases Faecal Abundance of Short-Chain Fatty Acid-Producing Bacteria and Microbiome Diversity: A Randomized, Controlled, Decentralized Trial

**DOI:** 10.3390/nu16070989

**Published:** 2024-03-28

**Authors:** Yanan Wang, Brooke Wymond, Himanshu Tandon, Damien P. Belobrajdic

**Affiliations:** 1 CSIRO, Microbiomes for One Systems Health-Future Science Platform, Health and Biosecurity, Adelaide 5000, Australia; yanan.wang@csiro.au; 2 CSIRO Health and Biosecurity, Adelaide 5000, Australia; brooke.wymond@csiro.au (B.W.); himanshu.tandon@csiro.au (H.T.)

**Keywords:** fibre, wholegrain, microbiome, diversity, short-chain fatty acid, butyrate, bread, gastrointestinal health

## Abstract

A low-fibre diet leads to gut microbiota imbalance, characterized by low diversity and reduced ability to produce beneficial metabolites, such as short-chain fatty acids (SCFAs). This imbalance is associated with poor gastrointestinal and metabolic health. We aimed to determine whether one dietary change, substitution of white bread with high-fibre bread, improves gut microbiota diversity and SCFA-producing capability. Twenty-two healthy adults completed a two-phase randomized, cross-over trial. The participants consumed three slices of a high-fibre bread (Prebiotic Cape Seed Loaf with BARLEYmax^®^) or control white bread as part of their usual diet for 2 weeks, with the treatment periods separated by a 4-week washout. High-fibre bread consumption increased total dietary fibre intake to 40 g/d, which was double the amount of fibre consumed at baseline or during the white bread intervention. Compared to white bread, the high-fibre bread intervention resulted in higher faecal alpha diversity (Shannon, *p* = 0.014) and relative abundance of the Lachnospiracae ND3007 group (*p* < 0.001, FDR = 0.019) and tended to increase the butyrate-producing capability (*p* = 0.062). In conclusion, substituting white bread with a high-fibre bread improved the diversity of gut microbiota and specific microbes involved in SCFA production and may enhance the butyrate-producing capability of gut microbiota in healthy adults. These findings suggest that a single dietary change involving high-fibre bread provides a practical way for adults to exceed recommended dietary fibre intake levels that improve gut microbiota composition and support gastrointestinal and metabolic health.

## 1. Introduction

Dietary fibre is recognized as playing a critical role in maintaining gastrointestinal health, and inadequate fibre intake is associated with a range of non-communicable diseases, such as coronary heart disease, stroke and type 2 diabetes [1]. Despite this, a significant proportion of people in many developed countries have dietary fibre intakes far lower than recommended daily amounts. Consistent with this, Australians consume on average 20 g of dietary fibre per day [2], which is well short of the 30 g or higher levels that have been shown to reduce the risk of developing diseases of the gastrointestinal tract, including colorectal cancer [3]. 

Although the amount of dietary fibre is important, so too is the type of fibre. Consuming a broad range of different fibres from a diverse range of minimally processed plant-based foods (wholegrain cereals, vegetables and fruit), particularly fermentable fibre, is important for shaping a healthy gut microbiota [4]. Furthermore, differences in fibre structure determine whether fibre is fermented by specific microbes, which can lead to functional benefits as opposed to simply assisting with laxation. The dominant fibres in the Australian diet are cellulose and hemi-cellulose from wheat, which are poorly fermented, whereas beta-glucans, fructans and resistant starches are consumed at much lower levels. The latter fibres are readily fermented by microbes that produce SCFAs, which have a broad range of beneficial effects locally and systemically. Butyrate, in particular, is a type of SCFA that is an important fuel for colonic epithelial cells; it strengthens gut barrier function and has been shown to have important immunomodulatory functions [5]. Furthermore, a decrease in faecal butyrate-producing bacteria has been reported in patients with inflammatory bowel disease, Crohn’s disease and type 2 diabetes [6,7,8]. Thus, dietary strategies that increase levels of butyrate-producing bacteria have potential for the prevention and/or treatment of these diseases.

As people in Australia and most developed countries continue to struggle to achieve recommended levels of fibre intake, there continues to be a strong need for foods high in a diverse range of fibres that can be readily incorporated into the diet. Bread and cereal products are the main dietary fibre sources for Australians, constituting 45% of their dietary fibre intake, followed by 10% from fruit and 30% from vegetables [9]. Subsequently, bread was chosen as the food for this study, as it is a staple, commonly consumed product in the Australian diet [10]. The bread included in the study was formulated to contain a high level of dietary fibre and a diverse number of plant-based ingredients that provide a range of fermentable fibres. One of the key ingredients in the bread is BARLEYmax^®^ (18%), and our group has previously shown that a diet high in BARLEYmax^®^-containing foods promoted faecal bulking, faecal total SCFAs and faecal butyrate levels [11,12]. However, it is not known whether simply replacing white bread in the diet with a high-fibre bread containing BARLEYmax^®^ and other fermentable cereal fibres can stimulate butyrate-producing bacteria and produce improvements in measures of gut health.

In the present study, we conducted a randomized, cross-over intervention to examine whether the consumption of a bread containing high levels of a diverse range of fibres (Prebiotic Cape Seed Loaf with BARLEYmax^®^) improved markers of gastrointestinal health in healthy Australian adults. White (wheat) bread was used as the control. The aim of the study was to determine if the substitution of white bread with Prebiotic Cape Seed Loaf with BARLEYmax^®^ resulted in higher levels of faecal SCFA-producing bacteria, greater microbial diversity and improved gut comfort. Changes in the butyrate-producing ability of the gut microbiota were evaluated by quantifying the gene contents of the key enzymes involved in the final step of butyrate synthesis, butyrate kinase and butyryl-CoA:acetate CoA-transferase [BCoAT] [13,14].

## 2. Materials and Methods

### 2.1. Study Population

A total of 26 healthy women and men (13 women, 9 men) were randomly assigned to the study (ACTRN 12622000535774). The inclusion criteria were as follows: aged 22–55 y; a parent or carer for at least one primary school-aged child aged 5 to 12 years; and understanding of the study requirements, including being willing to maintain body weight for the duration of the study (i.e., no more than 3 kg weight loss/gain) and collect stool samples, having access to a personal email inbox and smartphone, being prepared to adhere closely to the prescribed food consumption protocol, and being located within 15 km of Ashwood, Melbourne. The exclusion criteria were as follows: currently a smoker or vaper; currently pregnant or lactating; working night shifts; having a self-reported significant acute or chronic illness or any condition that may affect the applicant’s ability to participate in the study; having experienced a cardiovascular event, such as congestive heart failure, heart attack, stroke or angina (chest pain) in the 84 days prior to screening; currently having, or having a history of, inflammatory bowel disease (e.g., ulcerative colitis or Chron’s disease), coeliac disease, irritable bowel syndrome, chronic constipation or regular bouts of diarrhoea; a history of chemotherapy or radiotherapy treatment within the two years preceding screening; a self-reported alcohol intake exceeding 10 standard drinks per week on average over the 28 days preceding screening; having changed their usual dietary intake/pattern within the 28 days preceding screening; being on a weight-loss dietary pattern; having self-reported body weight fluctuations of more than 5 kg within the 3 months preceding screening; requiring concomitant treatment during the screening/baseline period with any medication that could influence the gastrointestinal tract (e.g., Loperamide); having used probiotics, prebiotic supplements, fibre supplements or antibiotics in the 28 days preceding screening; having participated in another research study within 30 days preceding the start of this study. Participants provided written, informed consent to the study protocol as approved by the Commonwealth Scientific and Industrial Research Organisation (CSIRO) Human Ethics Committee. This study was conducted between July and September 2022.

### 2.2. Recruitment and Screening

The participants were recruited by advertising through the CSIRO website, Facebook and targeted emails to people registered on the Bakers Delight mailing list who were located within 15 km of Ashwood, Victoria. To facilitate compliance, participants were provided with gift vouchers upon completion of the study to an amount corresponding to the time spent in the study.

**Figure 1:**
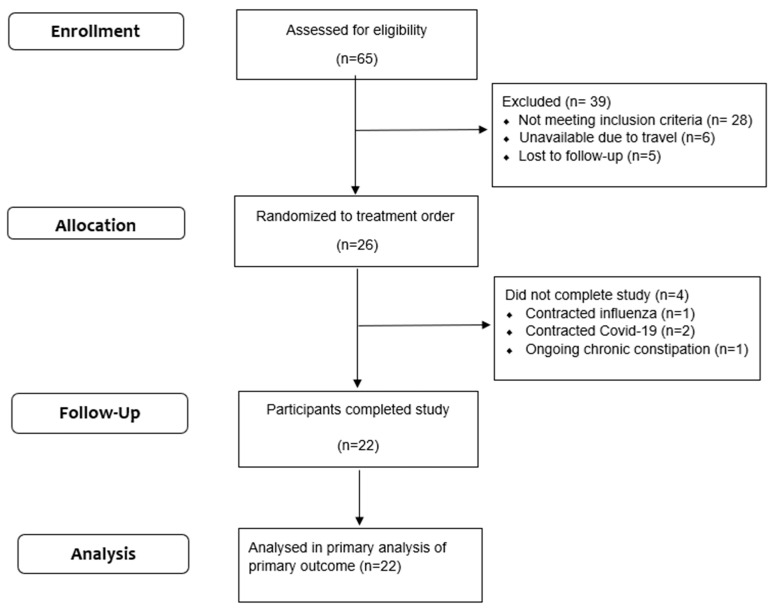
Participant flow.

Interested participants who responded to the study advertisements via email or telephone were contacted to determine eligibility. Participants were provided with information about the study design, and, if interested, a first screening telephone questionnaire was administered to determine general eligibility based on the study inclusion and exclusion criteria. Once eligibility was established, a pre-screening telephone appointment was scheduled to acquaint them with the study procedures. Sixty-five volunteers were screened, and twenty-six participants were enrolled in the study (Figure 1).

### 2.3. Study Design and Intervention

The study was designed as a single-centre, single-blinded, randomized, cross-over study that involved a random assignment of the order in which the participants received the two study treatments, which were high-fibre bread (Prebiotic Cape Seed Loaf with BARLEYmax^®^) and the control (white bread). The high-fibre bread was made from wholegrain wholemeal wheat flour (30%), water, BARLEYmax^®^ grain kibbled (18%), linseeds (6%), sesame seeds (6%), poppy seeds (4.5%), sunflower seeds (3%), wheat gluten, yeast, salt, barley malt, canola oil, soy flour, thiamin and folic acid. The ingredients for the white bread included wheat flour, water, iodized salt, yeast, vegetable oil (canola), soy flour and vitamins (thiamin and folic acid). The participants consumed the first assigned treatment bread product daily for 2 weeks, and this was followed by a 4-week washout period. They then consumed the second assigned treatment bread product for 2 weeks.

The study treatment bread products were delivered to the participants’ homes, and they were instructed to consume 3 × 50 g slices of the study bread each day. A dietitian provided options for the participants regarding how to include the study bread in their regular diet without making any major changes to their eating patterns. A short checklist was completed by the participants daily to aid their adherence to the study dietary protocol and was referred to when completing the weekly online survey. This weekly survey enabled us to determine whether a participant was complying with the study design.

Dietary intake was assessed at the beginning and the end of each treatment phase via a mobile app, and questionnaires were emailed and completed via the internet. Participants completed a daily log via a mobile app or paper-based diary to assess protocol compliance, adverse events and use of concomitant medications. Any queries that arose from the surveys were followed up by a phone call or email. In the 48 h period preceding days 0, 14, 42 and 56, participants provided a faecal sample for microbiome testing using a kit provided, and they visually assessed the faecal sample according to the Bristol stool rating system. Participants also completed a bowel-habit questionnaire. 

The CSIRO research team were blinded to the composition of each test bread. Although the study participants were provided the bread in unlabelled bags and were not informed of which bread product they were consuming, the type of bread was readily identifiable. Randomized allocation was conducted by the clinic manager according to an electronically generated simple randomization plan. Allocation concealment was conducted by the funder who provided the bread product to the study participants, and allocation details were stored by them in a sealed envelope. Treatment allocation was divulged once the data clean-up and preliminary statistical analysis had been completed by the project leader (DPB). 

The compositions of the bread products are shown in Table 1. The composition of the test bread was determined in duplicate using the following methods: moisture AOAC 930.15, ash AOAC 942.05, protein AOAC 992.23, fat AOAC 983.23, starch and resistant starch AOAC 2002.02, sugars AOAC 982.14, and total fibre and insoluble and soluble fibre AOAC 991.43. The total dietary fibre composition of the white bread was 2.7 g/100 g, providing 4.1 g fibre per day, and the high-fibre bread provided 15.3 g/100 g and 23.0 g fibre per day. The fibre provided by the high-fibre bread was predominately insoluble fibre (88%). The amount of resistant starch was very low for both test bread products.

The bread was delivered fresh on the day that it was baked. The participants were asked to store the loaves in their freezer and to thaw out slices as required over the two-week consumption period.

### 2.4. Twenty-Four-Hour Dietary Recall

Participants’ 24 h habitual dietary intake was estimated at the baseline and the endpoint of each phase using a digital food diary collected via a commercially developed smartphone application called Research Food Diary (RFD; Xyris Software, version 6, Brisbane, QC, Australia), which enables users to record food and beverage consumption. RFD has been shown to be valid compared to 24 h recalls and feasible and acceptable for use in research [15,16]. Once participants had been confirmed as eligible, they were given step-by-step written instructions on (1) downloading the RFD app onto their smartphone, (2) entering the required information into the app to sign up and enter diet intake information into the app, and (3) emailing the completed diet diaries to the CSIRO study staff. Each dietary recall was reviewed by the study dietitian to assess the completeness of the dietary information, and participants were contacted if any clarifications or additional details were required. Data from the app were directly uploaded to the Foodworks^®^ Professional dietary analysis software (Xyris Software Australia; using Australia’s largest food database, AusFoods) for nutrient analysis.

### 2.5. Digestive Comfort

Digestive comfort was assessed by a validated Gut Symptoms Rating Scale questionnaire [17]. This questionnaire asked the participants to provide a rating for upper gut symptoms (burping, belching, regurgitation, heartburn and nausea), general abdominal discomfort (abdominal pain, bloating and gurgling noises), lower gut symptoms (excessive gas, frequent bowel movements, urgent bowel motions and constipation), appetite (feelings of fullness, excessive hunger and ability to complete meals) and overall wellbeing. Participants were asked to rank each measure on a scale of 1–5 based on how often they had experienced a particular symptom in the previous 7 days (1, all of the time; 2, most of the time; 3, some of the time; 4, a little of the time; 5, never).

### 2.6. Faecal Sample Collection

Participants were provided with an EasySampler stool collection kit (GP Medical Devices, Holstebro, Denmark) to aid collection of a faecal sample, and they were instructed on how to collect a subsample into a Norgen tube (Norgen Biotek Corp., Thorold, ON, Canada). Following gentle shaking of the collection tubes, they were posted by the study participants to the analytical laboratory in Adelaide, South Australia, within 6.6 ± 2.1 (mean ± SD) days, where they were frozen at −80 until analyzed. 

The volunteers visually assessed each bowel motion according to the Bristol stool chart.

### 2.7. Microbiome Analysis

#### 2.7.1. Faecal DNA Extraction Method

Faecal samples were thawed, and DNA was extracted in singlet using the Qiagen DNAeasy 96 PowerSoil Pro (QIAcube HT Kit, Qiagen, Germantown, MD, USA) with the Tissue Lyser II beadmill (Qiagen, Germantown, MD, USA), as per the manufacturer’s instructions. Faecal sample extractions were performed in a random order; however, multiple visits for each individual were included within the same extraction run. 

#### 2.7.2. Microbiome Analysis 

16S rRNA amplicon sequencing targeting the V3V4 region was performed by the Australian Genome Research Facility (Brisbane, QL, Australia). PCR amplicons were generated using the primers and conditions outlined in the Supplementary Materials (Supplementary Methods). Thermocycling was completed with an Applied Biosystem 384 Veriti and using Platinum SuperFi II mastermix (Life Technologies, Australia) for the primary PCR, and the 16S sequencing was performed on an Illumina MiSeq (San Diego, CA, USA) with a V3, 600 cycle kit (2 × 300 base pairs (paired end)). Paired-end 16S rRNA gene amplicon sequence reads were analyzed with QIIME 2 (2019.7) [18]. Raw sequences were demultiplexed and trimmed for template-specific primers using cutadapt. Data were denoised, and amplicon sequence variants (ASVs) were generated using DADA2 within QIIME2 [19,20]. 

ASVs were classified taxonomically using the sklearn classifier using Silva (version 132) [21], which was pre-clustered at 99% identity. Alpha-diversity metrics (a measure of diversity within a gut microbiota community) and beta-diversity metrics (a measure of the similarity of two microbiota communities), weighted UniFrac, and unweighted UniFrac were estimated using q2-diversity. 

### 2.8. Quantification of the Faecal BCoAT Gene Content 

The number of copies of BcoAT genes was determined using quantitative polymerase chain reaction (qPCR), using the methods described by Louis et al. [13] with modifications. The PCR reaction was performed in a total volume of 10 µL using the SsoFast EvaGreen^®^ Supermixes (Bio-rad, Hercules, CA, USA) with 500 nM of each of the forward [GCNGANCATTTCACNTGGAAYWSNTGGCAYATG] and reverse primers [CCTGCCTTTGCAATRTCNACRAANGC] and 1 µL of DNA samples. Amplification and detection of DNA by real-time PCR were performed with the BioRad CFX384 Real Time System in duplicate. The reaction conditions for amplification were 98 °C for 2 min, 35 cycles of 98 °C for 10 s, 60 °C for 45 s, 72 °C for 45 s.

### 2.9. Statistical Analyses

Using clinical trial data from a dietary intervention study by Akagawa et al. (2021), it was estimated that a sample size of 21 would provide 80% power to detect a 3% increase in faecal butyric acid-producing bacteria. To allow for a potential dropout rate of up to 20% (*n* = 5), 26 participants were recruited into the study

The effects of the high-fibre and white bread on diet intake, gut comfort questionnaire and Bristol stool chart data were evaluated by determining the change from baseline (prior to the intake of the particular bread type), and the magnitude of change was determined using a paired Student’s *t*-test. The threshold for significance was set at 0.05 (two-sided). Statistical analyses were performed using SPSS software (IBM Corporation, New York, NY, USA, version 28.0.1.0).

Differences in alpha diversity (observed features, Shannon, Chao1 and Pielou’s evenness) within and between the treatment groups were assessed using the Wilcoxon signed-rank test (GraphPad Prism version 9.41). Differences in the beta diversity of microbiota were assessed using the permutational analysis of variance (PERMANOVA) model with 9999 permutations based on the parameters’ permutation of residuals under a reduced model and a type III sum of squares (Primer-E v.7; Primer-E Ltd., Auckland, New Zeland). Taxonomic differences at genus level were analyzed using the Wilcoxon test following the FDR procedure (Benjamini–Hochberg, R version 4.2.1). Graphs were made using R packages grafify [22].

## 3. Results

### 3.1. Participants’ Characteristics

Twenty-six healthy adults met the inclusion criteria for the study and were randomized. Four participants did not complete the study: one person contracted influenza, two people contracted COVID-19 and one person did not commence the intervention as they were experiencing chronic constipation (Figure 1). A total of 22 participants completed the cross-over study (9 men, 13 women). Faecal samples from the 22 participants were used for the 16S sequencing; however, due to sample loss, DNA samples from only 20 participants were used for the qPCR assay for quantifying the BCoAT gene levels. The study participants were 43 ± 5 years of age, weighed 72 ± 11 kg and had a body mass index of 24 ± 3 kg/m^2^. All data obtained from these individuals were analyzed.

### 3.2. Dietary Intake and Compliance

Study participant compliance in consuming three slices of the prescribed bread during both intervention periods was very high, with a mean intake of 3.0 ± 0.01 slices/d for each bread type. Dietary records showed that prior to each 2-week intervention period, the study participants consumed similar amounts of carbohydrate, starch and dietary fibre and servings of grain and refined grain (Table 2).

The high-fibre bread intervention increased the servings of wholegrains from one and a half to four per day and increased total dietary fibre intake to 40.1 g/d, which was double the amount of fibre consumed by the study participants at baseline or during the white bread intervention (Table 2). Study participants on the high-fibre bread intervention consumed fewer servings of refined grains and less starch but a similar amount of total carbohydrate compared to the white bread intervention (Table 2). 

### 3.3. Effect of High-Fibre Bread on Faecal Consistency

**Figure 2:**
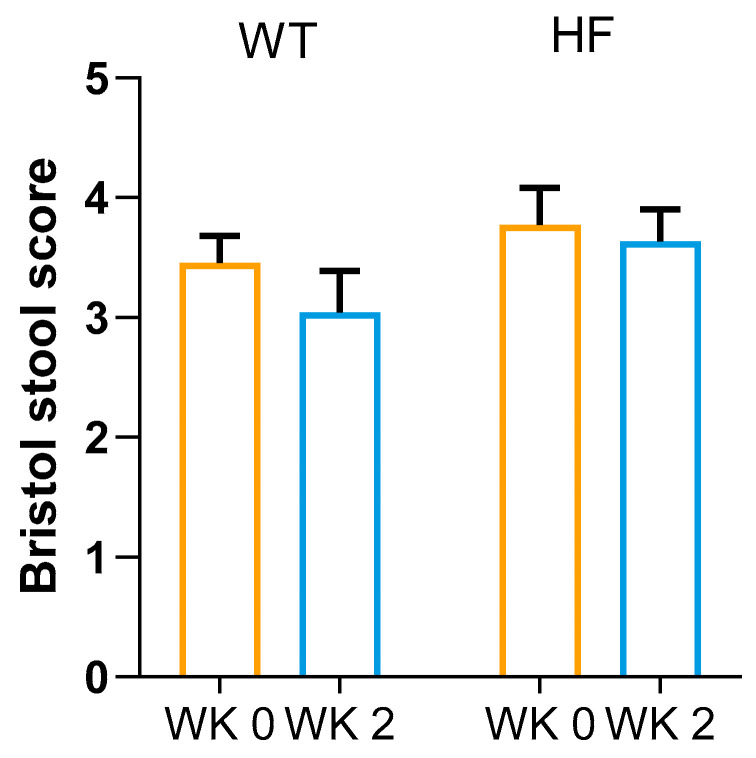
Bristol stool rating. Data are expressed as means ± SDs. Paired Student’s *t*-test (*n* = 22). WT, white bread; HF, high-fibre bread.

Prior to each bread intervention, the Bristol stool ratings were similar (Figure 2). The Bristol stool ratings remained similar to baseline levels when high-fibre or white bread was consumed (*p* = 0.220).

### 3.4. Effect of High-Fibre Bread on Gut Comfort

Throughout the study, the participants reported very low levels of gut discomfort, with average responses ranging from none (no symptoms) to slight, which is indicative of a healthy population with good gut health (Table S2). Consumption of the control or high-fibre bread products had no effect on upper or lower gastrointestinal symptoms, general abdominal discomfort, appetite, or general wellbeing (Table S2). Overall, the gut symptom scores were low for both dietary interventions and considered in the healthy range.

### 3.5. Effect of High-Fibre Bread on Faecal Microbial Diversity

**Figure 3:**
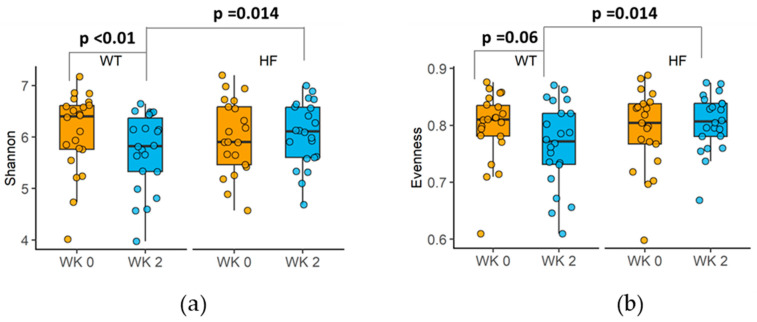
Changes in alpha diversity of faecal microbiota. (**a**) Changes in Shannon index. (**b**) Changes in Pielou’s evenness. Data presented as medians and inter-quartile ranges (*n* = 22). *p*-values from Wilcoxon signed-rank tests are displayed.

Following each 2-week intervention, the Shannon diversity index, a measure of microbial richness and evenness, was higher for the high-fibre bread compared to the white bread (median [IQR], HF 6.11 [5.60–6.60] vs. WT 5.82 [5.24–6.44], *p* = 0.014; Figure 3). Further analysis of richness and evenness independently showed that consumption of the high-fibre bread led to higher evenness (Pilou’s evenness) (median [IQR], HF 0.81 [0.76–0.84] vs. WT 0.77 [0.72–0.83], *p* = 0.014; Figure 3) compared to white bread, whereas richness (total number of different taxa, determined by the observed features index) was similar for the treatment groups (*p* = 0.69). Furthermore, within-treatment comparison of alpha diversity was different for white bread but not for high-fibre bread, suggesting that the difference in these alpha-diversity measures was primarily due to the reduction in alpha diversity observed for the white bread treatment group when the values were compared with the baseline (Figure 3). No group differences were observed at the endpoint for observed features and Chao1. 

The overall microbial composition (beta diversity), assessed based on weighted Unifrac distance, did not differ between the treatment groups (PERMANOVA *p* = 0.74) or within treatment groups (*p* = 0.97 for white bread and *p* = 0.86 for high-fibre bread).

### 3.6. Changes in Taxa following High-Fibre Bread Consumption 

**Figure 4:**
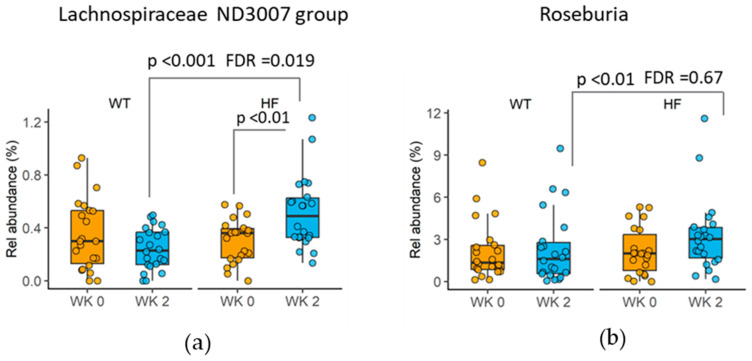
Differences in faecal microbiome taxa at genus level between treatment groups (endpoint differences with FDR-adjusted *p*-values < 1 are shown). (**a**) Changes in the relative abundance of the Lachnospiraceae ND3007 group. (**b**) Changes in the relative abundance of Roseburia. Data (relative abundance) presented as medians and inter-quartile ranges (*n* = 22). *p*-values were from Wilcoxon signed-rank tests and following adjustment for false discovery rate (FDR) (Benjamini–Hochberg).

Following the 2-week intervention, the high-fibre bread increased the relative abundance of Lachnospiracae ND3007 compared to white bread [high-fibre bread 0.5%, 0.3–0.7 and white bread 0.2%, 0.1–0.4 (median, inter-quartile range) (*p* < 0.001, FDR = 0.019; Figure 4a)] and tended to increase the relative abundance of Roseburia (*p* = 0.0074, FDR = 0.67; Figure 4b), a butyrate producer.

### 3.7. Effect of High-Fibre Bread on BCoAT Gene Content 

**Figure 5:**
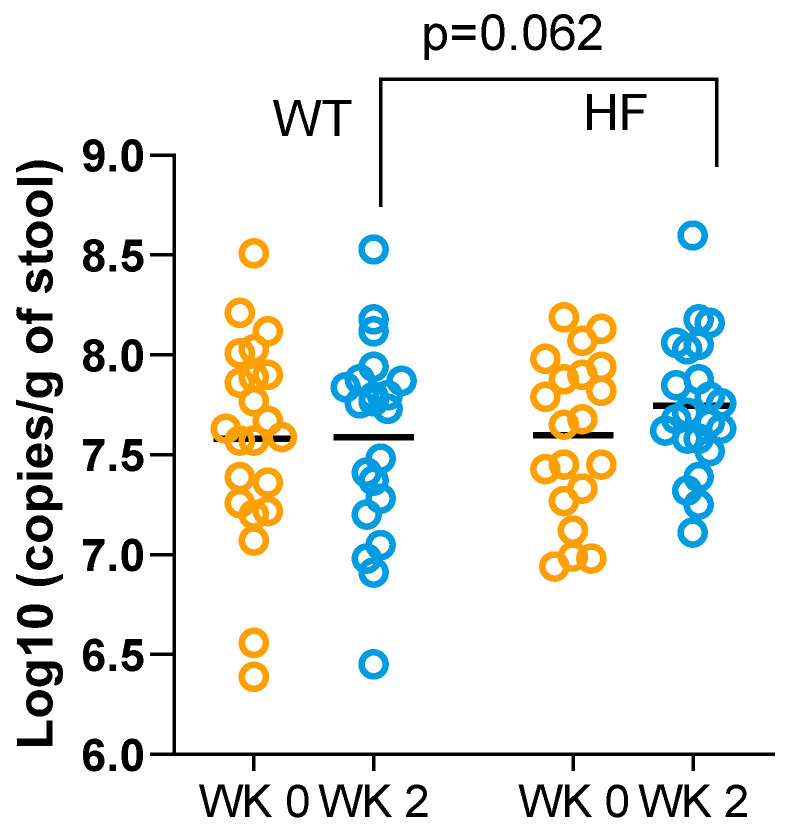
Changes in faecal gene content of butyryl-CoA:acetate CoA-transferase (BCoAT) following white bread and high-fibre bread consumption. Individual data and means are presented (*n* = 20). Paired *t*-tests.

To further investigate the effect of high-fibre bread with respect to increasing the butyrate-producing capability of the gut microbiota, we quantified the levels of BcoAT genes—marker genes for butyrate synthesis in the human colon. Extracted DNA from the faecal samples of 20 participants was used for the absolute quantification of BcoAT genes using qPCR. Again, the high-fibre bread tended to increase the level of BcoAT genes compared to white bread (Figure 5), but the group differences were not significant (mean ± SD, HF 7.05 ± 0.34 vs. WT 6.89 ± 0.49, log10 copies/g of stool, *p* = 0.062). 

## 4. Discussion

The current study evaluated whether a simple substitution of a commonly consumed food product in the diet of healthy adults could increase dietary fibre intake and improve gut microbiota diversity and SCFA-producing capability. Conventional white bread was replaced with Prebiotic Cape Seed Loaf with BARLEYmax^®^ containing high levels of fibre from cereals and seeds. The high-fibre bread intervention increased the servings of wholegrains from one and a half to four per day, doubled the amount of soluble fibre and increased total dietary fibre intake to 40 g/d, which was double the amount of fibre consumed by the study participants at baseline or during the white bread intervention. This increase in wholegrain and dietary fibre intake from a single dietary change of a commonly consumed food is striking, especially given that this dietary change is recognized as one of the most significant dietary changes associated with the reduction in death and disability-adjusted life years globally [23]. Overall, our study highlights a simple and feasible approach to reach or exceed the recommended level of daily fibre intake and promote gastrointestinal health. Incorporating three slices of high-fibre bread each day is a practical strategy to increase overall fibre intake, as bread is already a staple in the Australian diet. According to the most recent Australian National Health Survey (2011–2012), regular bread and bread rolls were the most commonly eaten food in the ‘grain (cereals)’ group, with a median intake in adults of 88 g, which is equivalent to just over two slices (around 40 g per slice) [24]. Adding one extra slice per day is likely to be easy to achieve for most people, as bread is a versatile food commonly enjoyed throughout the day, particularly at breakfast and lunch, as toast or as a sandwich. 

Consuming the high-fibre bread in the current study was associated with an increased faecal abundance of members of Lachnospiracae, in particular, the Lachnospiracae ND3007 group, which are carbohydrate-utilizing and putative SCFA-producing microbes [25]. This finding was more pronounced than the results of a study by Vanegas and colleagues, who only reported a trend for higher faecal abundance of *Lachnospira* when healthy adults consumed wholegrains (providing 40 g fibre/d) compared to the control group (no wholegrains and consuming 21 g fibre/day) [26]. In the current study, we also showed that, following high-fibre bread (3.0%, IQR: 1.5, 3.9) consumption, the abundance of Roseburia was nearly double the levels observed when white bread was consumed (1.6%, IQR: 0.5, 3.1), but this was not statistically significant after FDR correction. Consistent with this finding, we showed that the overall butyrate-producing ability of the faecal microbiota (BCoAT gene contents) tended to increase following the high-fibre bread compared to white bread consumption (*p* = 0.062). It is worth noting that samples from only 20 participants were available for the qPCR assay, which is slightly less than the sample size calculated for this study. Future studies with a larger sample size are needed to confirm whether high-fibre bread consumption enhances the abundance of Roseburia and the butyrate-producing capability of gut microbiota. Although the levels of resistant starch were not different between the two treatment breads, other fermentable fibres in BARLEYmax^®^, such as beta-glucan and fructans, may have contributed to enhancing the abundance of SCFA-producing bacteria. Changes in faecal butyrate-producing bacteria and/or SCFAs have been reported following the consumption of food products or diets containing BARLEYmax^®^. A recently published study showed that consumption of a granola containing BARLEYmax^®^ for 4 weeks increased the proportion of butyrate-producing bacteria (from 5.9% to 8.2%) and faecal butyric acid concentration (from 0.99 mg/g faeces to 1.43 mg/g after intake) [27]. We have previously reported higher faecal butyrate levels following the consumption of barley products in pigs and humans [11,12]. In particular, butyric, propionic and acetic acid levels were significantly higher in samples collected 48 h after the intake of BARLEYmax^®^ than in samples collected after the intake of wholewheat or refined cereal in these studies [12]. The high-fibre bread used in the current study contained 18% BARLEYmax^®^, and the daily consumed amount of BARLEYmax^®^ in the current study was similar to the level of barley consumed in previous studies. However, the shorter treatment period (2 weeks) of the current study may have limited the change in SCFA-producing bacteria compared to the two previous intervention studies, which had longer intervention periods of 4 weeks. 

It is also worth noting that a study providing a similar intervention to the current trial (150 g/d of bread containing a mixture of seven dietary fibres at two different levels of 5.55 g and 16.05 g/d) reported an increased abundance of SCFA/butyrate-producing microbes, *Parabacteroides distasonis* and *Fusicatenibacter saccharivorans* [27]. Overall, this finding is consistent with the current study in showing favourable changes in SCFA- and butyrate-producing bacteria, though the specific microbial species that changed in response to the intervention were different. These inter-study differences likely reflect the specificity of different SCFA-producing bacteria to specific types and mixtures of dietary fibres present in the different test breads used in the studies.

Faecal microbial diversity (alpha diversity) is recognized as an important measure of gastrointestinal health, with reduced diversity associated with higher disease risk, whereas higher alpha diversity is associated with healthy populations free from overt disease [28]. Although many differing dietary approaches to increase faecal microbial diversity have been explored, a narrative review reported that none of the eight higher-fibre randomized, controlled trials providing at least 28 g fibre/day showed an improvement in alpha diversity [29]. Additionally, a recent study that provided study participants with a granola containing BARLEYmax^®^ did not show a change in microbial diversity; however, the level of dietary fibre inclusion (5.7 g/d) was markedly lower than the level provided in the high-fibre bread arm of the current study (23 g/d). At this higher level of fibre consumption in the current study, faecal microbial alpha diversity was higher compared to white bread, but this difference was primarily due to the reduction in alpha diversity following white bread consumption for 2 weeks, whereas the alpha diversity remained unchanged following 2 weeks of consumption of the high-fibre bread. It is not clear why the white bread intervention caused this reduction in alpha diversity, as dietary fibre intake remained similar to baseline levels. Furthermore, the amount of total carbohydrate remained the same, yet the refined carbohydrate servings doubled, which suggests that the white bread was added to the existing refined carbohydrate foods yet compensated for by reduced intake of other non-cereal-based carbohydrate-rich foods, such as vegetables. This reduction in microbial diversity is of concern, as it is recognized as one of the main characteristics of dysbiosis, which is associated with many diseases and conditions, including inflammatory bowel disease [30], obesity [31] and type 2 diabetes [32]. Furthermore, group differences in alpha diversity in this study were observed for Shannon and Pielou’s evenness but not for richness alone (observed features), suggesting that evenness is more susceptible to dietary changes, a finding consistent with a recent review [33]. During adulthood, richness (the number of different taxa) is generally stable, and an increase in richness is rarely observed in in vivo studies involving diet changes [33]. On the other hand, when an intervention diet promotes the growth of a specific group of bacteria, evenness, reflecting the proportion of different taxa that make up the microbial community, may change. In the current study, high-fibre bread increased or tended to increase the relative abundance of members of Ruminococcaceae and Eubacterium, thereby impacting the distribution of taxa abundance.

This study was conducted on a healthy adult population who were consuming a low level of fibre in their background diet that was consistent with the typical fibre intake across the Australian population [2]. The high-fibre bread intervention markedly increased the level of fibre in the diet, which was well tolerated without adversely affecting digestive comfort.

The impact of high-fibre bread consumption on gut microbiota, including increased abundance of SCFA producers and potentially enhanced butyrate-producing ability, may provide significant benefits in maintaining intestinal homeostasis and protecting the host against inflammation-related intestinal diseases [34]. The protective function of butyrate against colorectal cancer and the anti-inflammatory mechanisms of butyrate within the intestinal tract have been well studied in vitro [34,35,36,37]. A human intervention study from Leu et al. [38] also reported that consumption of butyrylated high-amylose maize starch significantly increased butyrate and other SCFA levels, preventing red meat-induced adduct formation and thereby reducing risks of colorectal cancer. Beyond the gut health benefits, the effects of dietary fibre, particularly soluble fibre, in reducing risks of metabolic diseases, including diabetes and obesity, have been well demonstrated, either through or independently of altering the gut microbiota [39,40]. In the context of diabetes, increasing daily fibre intake by 15 g or to a target daily intake of 35 g, as achieved in the current study, is a target that is estimated to be associated with reduced risk of premature mortality in adults with diabetes [23]. Therefore, incorporating high-fibre bread with potential SCFA-promoting effects into the diet can be a practical and convenient strategy to fill the fibre gap, maintain gut health and support metabolic health. 

It is worth noting that the current study was conducted with a relatively short intervention period (2 weeks) and a small sample size (*n* = 22) in healthy middle-aged adults. We showed that the high-fibre bread intervention was effective in increasing gut microbial diversity and relative abundance of the Lachnospiracae ND3007 group compared to the white bread. However, a trend was only observed for higher abundance of a butyrate-producing microbe, Roseburia, and for higher overall butyrate-producing capacity (as measured by quantifying faecal BCoAT gene contents), which could be due to the short study duration and relatively small sample size. The high-fibre bread used in the current study contained a diverse mix of seeds and grains providing a range of fermentable fibres, including beta-glucan and fructans. Differing formulations of high-fibre breads and different sources of dietary fibre or even wholegrains could provide a more comprehensive understanding of the effects of dietary fibre on gut microbiota, particularly cereals containing high levels of resistant starches, which have been shown to favour butyrate-producing microbes [17,41,42,43]. Although the current study was limited to adults, further investigations in other populations struggling to meet recommended dietary fibre intakes, such as children and adolescents, are warranted. 

## 5. Conclusions

In conclusion, in healthy adults, the substitution of white bread with a high-fibre Prebiotic Cape Seed Loaf with BARLEYmax^®^ increased total dietary fibre intake to 40 g/d, which was double the amount of fibre consumed at baseline or during the white bread intervention. Compared to white bread, the high-fibre bread intervention resulted in higher faecal alpha diversity and relative abundance of the Lachnospiracae ND3007 group and tended to increase the butyrate-producing capability. This provides a simple and readily achievable approach for adults to reach and exceed the recommended intakes of dietary fibre and wholegrains. It also supports the need for larger, longer-term interventions with greater sample sizes to further evaluate the potential gastrointestinal and related metabolic health benefits of this product for children and adults.

## Figures and Tables

**Table 1:** Nutrient contents of treatment breads ^1^.

	White Bread ^2^		High-Fibre Bread ^3^	
	Per 100 g	Per 3 Slices (114 g)	Per 100 g	Per 3 Slices (150 g)
Energy (kJ)	1080	1231	1240	1860
Protein (g)	9.5	10.8	14.8	22.2
Fat (total) (g)	1.9	2.2	10.6	15.9
Carbohydrate (g)	54.0	61.6	24.7	37.1
Starch	50.5	57.6	22.0	33.0
Sugars (g)	3.5	4.0	2.7	4.1
Total dietary fibre (g)	2.7	3.1	15.3	23.0
Resistant starch	0.6	0.7	0.5	0.8
Insoluble fibre (g)	1.5	1.7	14.6	21.9
Soluble fibre (g)	1.3	1.5	2.1	3.2

^1^ Mean values are reported for samples analyzed in duplicate. ^2^ White block loaf (Bakers Delight, Ashwood, VIC, Australia). ^3^ Prebiotic Cape Seed Loaf with BARLEYmax^®^ (Bakers Delight, Ashwood, VIC, Australia).

**Table 2:** Daily energy and nutrient intake.

	White Bread			High-Fibre Bread			
	WK 0	WK 2	∆	WK 0	WK 2	∆	*p*-Value
Energy (kJ)	8576 ± 2494	9309 ± 2418	732 ± 3100	8177 ± 2838	8333 ± 1909	157 ± 2626	0.601
Carbohydrate (g)	222.4 ± 76.9	249.2 ± 83.4	27 ± 108	196.3 ± 65.3	185.9 ± 46.4	−10 ± 53	0.263
Starch (g)	128.3 ± 51.8	185.9 ± 67.6	57 ± 71	114.1 ± 37.2	104.1 ± 33.8	−10 ± 33	0.008
Sugars (g)	93.3 ± 56.6	67.3 ± 25.9	−26 ± 52	80.3 ± 43.9	73.9 ± 27.2	−6 ± 35	0.189
Total dietary fibre (g)	21.9 ± 10.7	20.3 ± 8.1	−2 ± 13	19.0 ± 7.2	40.1 ± 6.3	21 ± 9	<0.001
Grain (servings)	5.5 ± 3.6	9.8 ± 4.5	4.3 ± 4.3	5.6 ± 1.9	6.1 ± 2.1	0.5 ± 2.8	0.026
Refined grain (servings)	4.4 ± 3.5	9.3 ± 4.0	4.9 ± 4.0	4.1 ± 2.0	4.1 ± 2.0	0 ± 0	<0.001
Wholegrain (servings)	1.1 ± 1.3	0.5 ± 1.0	−0.6 ± 1.6	1.5 ± 1.5	4.0 ± 0.5	2.5 ± 1.6	<0.001

Data are expressed as means ± SDs. Change data compared by a paired Student’s *t*-test (*n* = 22).

## Data Availability

The data presented in this study are available on request from the corresponding author.

## Supplementary Materials

The following supporting information can be downloaded at: https://www.mdpi.com/article/10.3390/nu16070989/s1, Table S1: PCR primer conditions; Table S2: Perceived feelings of gut comfort, appetite and general well-being.
