# Supplementary material for: Swapping White for High-Fibre Bread Increases Faecal Abundance of Short-Chain Fatty Acid-Producing Bacteria and Microbiome Diversity: A Randomized, Controlled, Decentralized Trial
Source: Nutrients. 2024 Mar 28;16(7):989. doi: 10.3390/nu16070989 (PMC11013647; doi:10.3390/nu16070989)
Supplement: Supplementary file 1 [file nutrients-16-00989-s001.zip › nutrients-2878649-supplementary.pdf]

Supplementary Materials

Swapping White for High-Fibre Bread Increases Faecal Abundance of Short-Chain Fatty Acid-Producing Bacteria and Microbiome Diversity: A Randomized, Controlled, Decentralized Trial

Yanan Wang, Brooke Wymond, Himanshu Tandon and Damien P. Belobrajdic *

CSIRO, Microbiomes for One Systems Health-Future Science Platform, Health and Biosecurity, Adelaide 5000, Australia; yanan.wang@csiro.au (Y.W.); brooke.wymond@csiro.au (B.W.); himanshu.tandon@csiro.au (H.T.)

* Correspondence: damien.belobrajdic@csiro.au; Tel.: +61-8-8303 8815

Supplementary Methods

PCR primers and condition for 16S rRNA amplicon sequencing

PCR amplification and sequencing was performed by the Australian Genome Research Facility. PCR amplicons were generated using the primers as follow: forward primer(341F) CCTAYGGGRBGCASCAG
and reverse primer(806R) GGACTACNNGGGTATCTAAT. The conditions of PCR outlined in Table S1. Thermocycling was completed with an Applied Biosystem 384 Veriti and using Platinum SuperFi II mastermix (Life Technologies, Australia) for the primary PCR. The first stage PCR was cleaned using magnetic beads, and samples were visualised on 2% Sybr Egel (Thermo-Fisher). A secondary PCR to index the amplicons was performed with Platinum SuperFi II mastermix (Life Technologies, Australia). The resulting amplicons were cleaned again using magnetic beads, quantified by fluorometry (Promega Quantifluor) and normalised. The eqimolar pool was cleaned a final time using magnetic beads to concentrate the pool and then measured using a High-Sensitivity D1000 Tape on an Agilent 2200 TapeStation. The pool was diluted to 5nM and molarity was confirmed again using a Qubit High Sensitivity dsDNA assay (ThermoFisher). This was followed by sequencing on an Illumina MiSeq (San Diego, CA, USA) with a V3, 600 cycle kit (2 x 300 base pairs paired-end).

Table S1. PCR primer conditions.

Target	Cycle	Initial	Disassociate	Anneal	Extension	Finish
16S: V3 - V4	30	98 *C for 30 sec	98*C for 10 sec	60*C for 10 sec	72*C for 30 sec	72*C for 5 min

Supplementary Results

Table S2. Perceived feelings of gut comfort, appetite and general well-being [1].

	Week	White bread	High fibre bread	P value
Bristol stool rating	0	3.5 ± 0.2	3.6 ± 0.3	
	2	3.0 ± 0.3	3.6 ± 0.3	
	Δ	-0.4 ± 1.9	-0.1 ± 1.0	0.220
Have you been bothered by stomach ache or pain during the past week? (Stomach ache refers to all kinds of aches or pains in your stomach or belly.)	0	1.4 ± 0.2	1.5 ± 0.2	
	2	1.7 ± 0.2	2.0 ± 0.3	
	Δ	0.4 ± 0.9	0.5 ± 0.8	0.591
Have you been bothered by heartburn during the past week? (By heartburn we mean a burning pain or discomfort behind the breastbone in your chest.)	0	1.3 ± 0.3	1.0 ± 0.05	
	2	1.2 ± 0.1	1.2 ± 0.2	
	Δ	-0.2 ± 0.7	0.2 ± 1.2	0.358
Have you been bothered by acid reflux during the past week? (By acid reflux we mean regurgitation or flow of sour or bitter fluid into your mouth.)	0	1.3 ± 0.2	1.1 ± 0.1	
	2	1.3 ± 0.2	1.3 ± 0.3	
	Δ	0.1 ± 0.7	0.2 ± 1.1	0.659
Have you been bothered by hunger pains in the stomach or belly during the past week? (This hollow feeling in the stomach is associated with the need to eat between meals.)	0	1.3 ± 0.1	1.5 ± 0.2	
	2	1.4 ± 0.1	1.3 ± 0.2	
	Δ	0.2 ± 0.7	-0.3 ± 0.9	0.134
Have you been bothered by nausea during the past week? (By nausea we mean a feeling of wanting to be sick.)	0	1.2 ± 0.1	1.3 ± 0.2	
	2	1.3 ± 0.2	1.1 ± 0.1	
	Δ	0.1 ± 0.6	-0.2 ± 0.8	0.230
Have you been bothered by rumbling in your stomach or belly during the past week? (Rumbling	0	1.3 ± 0.1	1.6 ± 0.2	
	2	1.5 ± 0.2	1.7 ± 0.2	

refers to vibrations or noise in the stomach				
	Δ	0.3 ± 0.7	0.1 ± 0.9	0.379
Has your stomach felt bloated during the past week? (Feeling bloated refers to swelling in the stomach or belly.)	0<br>2	1.6 ± 0.2<br>2.0 ± 0.3	1.8 ± 0.2<br>2.1 ± 0.2	
	Δ	0.5 ± 1.3	0.3 ± 0.9	0.592
Have you been bothered by burping during the past week? (Burping refers to bringing up air or gas through the mouth.)	0<br>2	1.2 ± 0.1<br>1.3 ± 0.1	1.4 ± 0.2<br>1.3 ± 0.1	
	Δ	0.1 ± 0.4	-0.1 ± 0.6	0.359
Have you been bothered by passing gas or flatus during the past week? (Passing gas or flatus refers to the release of air or gas from the bowel.)	0<br>2	1.7 ± 0.2<br>1.8 ± 0.2	1.8 ± 0.2<br>2.4 ± 0.3	
	Δ	0.1 ± 0.7	0.5 ± 1.1	0.143
Have you been bothered by constipation during the past week? (Constipation refers to a reduced ability to empty the bowels.)	0<br>2	1.4 ± 0.1<br>1.5 ± 0.2	1.7 ± 0.3<br>1.5 ± 0.2	
	Δ	-0.1 ± 0.9	-0.1 ± 0.9	1.0
Have you been bothered by diarrhoea during the past week? (Diarrhoea refers to frequent loose or watery stools.)	0<br>2	1.4 ± 0.2<br>1.2 ± 0.1	1.3 ± 0.1<br>1.4 ± 0.3	
	Δ	-0.2 ± 0.4	0.1 ± 0.8	0.262
Have you ever been bothered by loose stools during the past week? (If your stools have been alternately hard and loose, this question only refers to the extent you have been bothered by the stools being loose.)	0<br>2	1.5 ± 0.2<br>1.3 ± 0.1	1.5 ± 0.2<br>1.5 ± 0.3	
	Δ	-0.3 ± 0.6	0.1 ± 0.9	0.249
Have you been bothered by hard stools during the past week? (If your stools have been alternately	0<br>2	1.6 ± 0.2<br>1.5 ± 0.2	1.5 ± 0.2<br>1.2 ± 0.1	

hard and loose, this question only refers to the extent you have been bothered by the stools being hard.)				
	Δ	-0.1 ± 1.1	-0.1 ± 0.6	1.0
Have you been bothered by an urgent need to have a bowel movement during the past week? (This urgent need to open your bowels makes you rush to the toilet.)	0	1.4 ± 0.2	1.4 ± 0.2	
	2	1.4 ± 0.2	1.6 ± 0.3	
	Δ	-0.1 ± 0.6	0.2 ± 0.8	0.330
When going to the toilet during the past week, have you had the feeling of not completely emptying your bowels? (The feeling that after finishing a bowel movement, there is still more stool that needs to be passed.)	0	1.9 ± 0.2	2.0 ± 0.3	
	2	2.0 ± 0.2	2.0 ± 0.3	
	Δ	0.1 ± 0.7	0.2 ± 1.7	0.755
Gut symptom score[2]	0	21.4 ± 1.6	22.4 ± 1.6	
	2	22.4 ± 1.5	23.7 ± 2.5	
	Δ	0.9 ± 3.1	1.6 ± 6.8	0.662

[1]Data are expressed as mean ± SD. Change data compared by a paired student t-test (n=20).

[2]Gut symptom score is sum of all scores. Discomfort scores were rated as 1 None, 2 slight, 3 mild, 4 moderate, 5 moderately severe, 6 severe, 7 Very severe discomfort.
